# CXCR4 PET imaging of mantle cell lymphoma using [^68^Ga]Pentixafor: comparison with [^18^F]FDG-PET

**DOI:** 10.7150/thno.48620

**Published:** 2021-01-01

**Authors:** Marius E. Mayerhoefer, Markus Raderer, Wolfgang Lamm, Verena Pichler, Sarah Pfaff, Michael Weber, Barbara Kiesewetter, Markus Hacker, Lukas Kazianka, Philipp B. Staber, Hans-Juergen Wester, Johannes Rohrbeck, Ingrid Simonitsch-Klupp, Alexander Haug

**Affiliations:** 1 Dept. of Biomedical Imaging and Image-guided Therapy, Division of General and Pediatric Radiology, Medical University of Vienna, Austria; 2 Department of Radiology, Memorial Sloan Kettering Cancer Center, New York, USA; 3 Dept. of Internal Medicine I, Division of Oncology, Medical University of Vienna; 4 Dept. of Biomedical Imaging and Image-guided Therapy, Division of Nuclear Medicine, Medical University of Vienna, Austria; 5 Dept. of Internal Medicine I, Division of Hematology and Hemostaseology, Medical University of Vienna; 6 Pharmaceutical Radiochemistry, Technical University of Munich, Garching, Germany; 7 Clinical Institute of Pathology, Medical University of Vienna

**Keywords:** Lymphoma, Chemokine receptor, CXCR4, Positron emission tomography, Magnetic resonance imaging

## Abstract

For PET imaging of mantle cell lymphoma (MCL), [^18^F]FDG (2-deoxy-2-[^18^F]fluoro-D-glucose) is the currently recommended radiotracer, although uptake is variable and bone marrow evaluation is limited. In this prospective study, we evaluated the novel CXCR4 (G-protein-coupled C-X-C chemokine receptor type 4) tracer [^68^Ga]Pentixafor in MCL patients, and compared it to [^18^F]FDG.

**Methods:** MCL patients underwent [^68^Ga]Pentixafor-PET/MRI, and, if required for routine purposes, also [^18^F]FDG-PET/MRI, before treatment. PET was evaluated separately for 23 anatomic regions (12 lymph node stations and 11 organs/tissues), using MRI as the main reference standard. Standardized uptake values (SUV_max_ and SUV_mean_) and tumor-to-background ratios (TBR_blood_ and TBR_liver_) were calculated. General Estimation Equations (GEE) were used to compare [^68^Ga]Pentixafor-PET and [^18^F]FDG-PET sensitivities and positive predictive values (PPV). For bone marrow involvement, where biopsy served as the main reference standard, and splenic involvement, receiver operating characteristic curves were used to determine the optimal SUV and TBR cut-off values, and areas under the curve (AUC) were calculated.

**Results:** Twenty-two MCL patients were included. [^68^Ga]Pentixafor-PET sensitivity (100%) was significantly higher than for [^18^F]FDG-PET (75.2%) (*P*<0.001), and PPV was slightly, but not significantly lower (94.0%.vs. 96.5%; *P*=0.21). SUVs and TBRs were significantly higher for [^68^Ga]Pentixafor-PET than for [^18^F]FDG-PET (*P*<0.001 in all cases); the greatest difference was observed for mean TBR_blood_, with 4.9 for [^68^Ga]Pentixafor-PET and 2.0 for [^18^F]FDG-PET. For bone marrow involvement, [^68^Ga]Pentixafor-PET SUV_mean_ showed an AUC of 0.92; and for splenic involvement, TBR_blood_ showed an AUC of 0.81.

**Conclusion:** [^68^Ga]Pentixafor-PET may become an alternative to [^18^F]FDG-PET in MCL patients, showing clearly higher detection rates and better tumor-to-background contrast.

## Introduction

Mantle cell lymphoma (MCL) is one of the five most common types of Non-Hodgkin lymphoma 1. The clinical course of MCL is variable -while in most cases it presents with a rapid, aggressive course, it manifests as a slowly growing, indolent disease in few patients 2. Apart from lymph node involvement, the spleen, bone marrow, and gastrointestinal tract are common sites of MCL. Despite the availability of novel types of treatment, the prognosis for MCL patients is generally considered to be poor, with 5-year survival rates of only about 50% 2,3.

Positron emission tomography (PET) using the “standard” radiotracer [^18^F]FDG (2-deoxy-2-[^18^F]fluoro-D-glucose) is officially recommended for the staging of MCL 4. However, since the glucose metabolism of MCL, and thus, [^18^F]FDG uptake, is sometimes only low-to-moderate 5, investigation of other PET radiotracers for use in MCL patients is justified.

It has been previously shown that MCL expresses high levels of the G-protein-coupled C-X-C chemokine receptor type 4 (CXCR4) 6. CXCR4 is activated through its ligand CXCL12, which, in turn, activates the mitogen-activated protein kinase and phosphoinositide 3-kinase pathway. In MCL, as well as other hematological malignancies, such as chronic lymphocytic leukemia and myeloma, CXCR4/CXCL12 is known to mediate tumor cell migration (“homing”) and adhesion to bone marrow stromal cells, which function as a protective microenvironment 6-9. Consequently, CXCR4 silencing in MCL cells has been shown to lead to a significant reduction in proliferation, decreased cell adhesion to bone marrow stromal cells, and formation of fewer colonies 10. Thus, the CXCR4/CXCL12 pathway represents a clinically interesting target for treatments such as ibrutinib, which is an inhibitor of Bruton's tyrosine kinase (BTK) 10, a key player in B-cell receptor signaling 11. Ibrutininb was approved as a second-line treatment for MCL by the FDA in 2013. The CXCR4 antagonist Plerixafor, which can be used for stem cell mobilization in patients with Non-Hodgkin lymphomas, including MCL, who respond poorly to granulocyte colony-stimulating factor alone, was approved by the FDA even earlier, in 2008 12.

Recently, [^68^Ga]Pentixafor has become available as a new PET tracer that specifically targets the CXCR4 receptor 13,14, and can thus be applied to lymphoproliferative diseases. Initial studies in multiple myeloma, acute and chronic leukemias, and selected Non-Hodgkin lymphomas have yielded encouraging results 15-22. However, no dedicated evaluation of [^68^Ga]Pentixafor-PET in MCL patients has been performed so far in MCL patients.

The aim of the present, prospective PET/MRI study was, therefore, to investigate whether whole-body CXCR4 imaging of MCL using [^68^Ga]Pentixafor-PET is feasible for the assessment of disease burden in this lymphoma subtype, and also to compare it to the clinical standard, [^18^F]FDG-PET. Since no imaging technique is presently established for the assessment of bone marrow involvement in MCL 4, it was also of interested to evaluate [^68^Ga]Pentixafor regarding this unmet diagnostic need. Finally, because the spleen shows a non-negligible physiologic [^68^Ga]Pentixafor uptake 18,19,22, we aimed to determine a cut-off value for the diagnosis of MCL involvement.

## Methods

### Patients and design

Treatment-naïve patients with MCL were enrolled in this prospective, proof-of-concept [^68^Ga]Pentixafor-PET/MRI study. The study was approved by the Ethics Committee of the Medical University of Vienna. Written, informed consent was obtained from all patients. Inclusion criteria were: histological verification of MCL through biopsy samples analyzed by a reference pathologist, according to the 2016 revision of the World Health Organization classification of lymphoid neoplasms; the ability of patients to understand the study goals or outline; and the ability to give written, informed consent. Exclusion criteria were: clinically confirmed pregnancy for women (e.g., by clinical examination, ultra-sound, or a pregnancy test); breast-feeding women; age below the specified minimum of 18 years; known contraindication to MRI (e.g., implantable medical devices according the MRI Safety Guidelines, or conditions such as claustrophobia); and, for patients who also underwent [^18^F]FDG-PET/MRI for routine clinical purposes, known diabetes and a blood glucose level of >150 mg/dL (>8.33 mmol/L) for this additional examination. PET/MRI was chosen over PET/CT (which, at our institution, is routinely performed with full-dose diagnostic, contrast-enhanced CT) to minimize radiation burden for patients undergoing imaging with both [^68^Ga]Pentixafor and [^18^F]FDG while being able to apply the same (MRI-based) PET attenuation correction technique.

### Radiotracer production

[^68^Ga]Pentixafor was synthesized using a fully automated Scintomics GRP+ module. The eluate of a ^68^Ge/^68^Ga-generator (GalliaPharm, EZAG, Germany) was concentrated using a Chromafix PS-H^+^ and heated with a solution of 40 µg Pentixafor (unlabeled precursor; GMP quality) in 1.5 mL of HEPES buffer (N-(2-hydroxyethyl)piperazine-N´-(2-ethanesulfonic acid), 1.5 M at 125°C for six minutes. Unbound and colloidal gallium-68 was removed using a SepPak Light C18 cartridge (Waters). The purified product was formulated with PBS buffer. The chemical and radiochemical purity of [^68^Ga]Pentixafor were analyzed by high performance liquid chromatography (HPLC) using an analytic Chromolith® Performance, RP-18e, 100-4.6 mm from Merck (Germany). Free ^68^Ga^3+^-ions and ^68^Ga-colloid were detected using radio-thin-layer chromatography (radio-TLC).

The glucose analogue [^18^F]FDG was synthesized using FASTlab FDG cassettes with a phosphate buffer formulation and a GE FASTlab platform (GE Healthcare).

### Imaging protocol

All PET/MRI examinations were performed on a fully CE-certified, integrated, simultaneous hybrid system (mMR; Siemens, Erlangen, Germany). The PET/MRI system is made of a PET detector system inserted into a 3T MRI system with high-performance gradient systems (45 mT/m) and a slew rate of 200 T/m/s. It is equipped with Total Imaging Matrix coil technology (Siemens), covering the body (from the vertex to the upper thigh) with multiple integrated radiofrequency surface coils. For PET, the system offers an axial FOV of 258 mm, and a sensitivity of 13.2 cps/kBq.

[^68^Ga]Pentixafor-PET/MRI was performed 60 min after the intravenous administration of 150 MBq of [^68^Ga]Pentixafor, with 5 min per bed position, four iterations and 21 subsets, a 4.2-mm slice thickness, and a 172x172 matrix, using the point-spread function-based reconstruction algorithm HD-PET (Siemens, Erlangen, Germany). For patients who also underwent [^18^F]FDG-PET/MRI before treatment initiation, this test was performed within a maximum of seven days from [^68^Ga]Pentixafor-PET/MRI, with the same PET acquisition parameters as the latter, 60 min after intravenous administration of 3 MBq/kg body mass of [^18^F]FDG. Patients had fasted for at least five hours prior to [^18^F]FDG injection.

For both [^68^Ga]Pentixafor-PET/MRI and [^18^F]FDG-PET/MRI, a two-point Dixon, 3D volume-interpolated breath-hold (VIBE) T1-weighted sequence was acquired for attenuation correction (AC) using the following parameters: repetition time (TR)/echo times (TE) 3.6/TE1=1.23 ms, TE2=2.46 ms; one average, two echoes; a 10° flip angle; a 320x175 matrix with a 430x309 mm FOV; and a 3-mm slice thickness with a 0.6-mm gap. A coronal T2-weighted half-Fourier acquisition single-shot turbo spin-echo (HASTE) sequence was acquired with a TR/TE of 1400/121 ms; a 160° flip angle; a 256x256 matrix with a 380x380 mm FOV; and a 6-mm slice thickness with a 1.2 mm gap. Finally, an axial, echo-planar imaging (EPI), spectral adiabatic inversion recovery diffusion-weighted imaging (DWI) sequence was obtained during free-breathing, with b-values of 50 and 800, using a TR/TE of 6800/63 ms; a 180° flip angle; a 440x340 matrix with a 168x104 mm FOV; and a 6-mm slice thickness with a 1.2 mm gap. Apparent diffusion coefficient (ADC) maps were generated.

### Image analysis

[^68^Ga]Pentixafor-PET was read with the raters blinded to the MRI component (with exception of the MRI-based attenuation correction maps that provided low-resolution anatomic information), to patients' reports from clinical practice (e.g., biopsy, surgery, and pathology, clinical examinations) and other imaging data or reports, in random order. In patients who also underwent [^18^F]FDG-PET/MRI, we followed the same strategy for [^18^F]FDG-PET image analysis; the minimum time interval between evaluations of [^68^Ga]Pentixafor-PET and [^18^F]FDG-PET was two weeks.

Independently for [^68^Ga]Pentixafor-PET and [^18^F]FDG-PET, and blinded to the respective other PET, raters had to decide and annotate which of the following 12 lymph node stations were positive for lymphoma, based on pathological tracer accumulations: right and left cervical (including supraclavicular, occipital, and preauricular nodes); right and left axillary (including subpectoral and infraclavicular); mediastinal (including mammary nodes); hilar; retroperitoneal; mesenteric; right and left pelvic; and right and left inguinal. In addition, extranodal involvement was assessed for the following 11 organs/tissues, again based on focal uptake on PET: Waldeyer ring; lungs; liver; pancreas, stomach, small intestine, large intestine, adrenal glands, kidneys, soft tissues (skin/fat/ muscle); and other organs/tissues (e.g., salivary glands and glandular breast tissue). For each involved nodal and extranodal site, maximum and mean standardized uptake values (SUV_max_ and SUV_mean_) were measured based on isocontour volumes of interest generated using the 41% SUV_max_ threshold previously recommended for [^18^F]FDG-PET 23, to enable a fair comparison between the two tracers. SUVs were also measured for a 3-cm³ spherical VOI that was manually placed in the liver, and a 1-cm³ spherical VOI placed in the aortic arch, which were used as reference tissues for the calculation of tumor-to-background ratios (TBR_liver_ and TBR_blood_: lesion SUV_max_ / reference tissue SUV_mean_).

Since no physiologic cut-off values or reference tissues are presently established for splenic and bone marrow [^68^Ga]Pentixafor uptake, they were evaluated separately. For the spleen, SUV calculations were based on a 3-cm³ spherical VOI that was placed in the center of the organ, and for the bone marrow, SUVs were extracted from a metabolic tumor volume that covered the bony pelvis.

Following their independent evaluation, the annotated [^68^Ga]Pentixafor-PET and [^18^F]FDG-PET images were reviewed side-by-side, and respective maximum transaxial lesion diameters were recorded on the co-registered MRI component for each anatomic site (i.e., lymph node station or organ/tissue) that was rated as positive on [^68^Ga]Pentixafor-PET and/or [^18^F]FDG-PET. For the spleen, the maximum vertical organ diameter was measured on MRI, as recommended in the Lugano guideline 4. For the bone marrow, the presence of marrow involvement was assessed on DWI and T1-weighted MRI as described in the recently published MY-RADS classification for multiple myeloma 24. In short, focal lesions or diffuse changes with signal intensity greater than muscle on b-800 DWI and hypointense appearance on T1, and/or ADCs of 700-1400 μm²/s were rated as positive for lymphoma involvement.

### Reference standard

MRI and biopsies served as the basis of the reference standard for all anatomic sites except the spleen and the bone marrow (see below). For confirmation of PET-positive findings, biopsy or (in the majority of cases) a corresponding lesion on DWI and T1- or T2-weighted MRI was required, and, in case of lymph nodes, also a long axis diameter of >1.5 cm on axial MRI, in accordance with the Lugano classification 4.

For the spleen, a positive biopsy result, or fulfillment of at least two of the following imaging criteria, was required to confirm lymphoma involvement: splenomegaly with a vertical spleen diameter >13 cm on coronal MRI, as recommended by the Lugano classification 4; a diffusely increased [^18^F]FDG uptake higher than that of the liver 25,26; one or more [^18^F]FDG-avid focal lesions and/or lesions with diffusion restriction on DWI, corresponding to focal uptake on [^68^Ga]Pentixafor-PET.

For confirmation of bone marrow involvement, unilateral iliac crest biopsy (i.e., histology, complemented by flow cytometry and/or fluorescence in situ hybridization analyses, where available) was the main reference standard, in accordance with routine clinical practice 4. When biopsy results were not available, both increased [^18^F]FDG-PET uptake (visually higher than the liver background) 19,27 and an MRI correlate according to MY-RADS were required 24.

### Immunohistochemistry

Immunohistochemistry (IHC) with an antibody against CXCR4 (Epitomics, Burlingame, CA) was done on 4 μm paraffin sections with a LEICA Bond III fully automated staining system, using the Bond Polymer Refine detection system and reagents supplied by Leica Microsystems, Newcastle-Upon-Tyne, UK, as recently described 28. Double-labeling of CXCR4 and Cyclin D1 for exact demonstration of staining of the lesional MCL cells was performed with a sequential double-staining method on the LEICA-BOND system, with CXCR4 as the first antibody visualized in brown, followed by Cyclin D1 incubation providing a nuclear red staining signal. Percentages of CXCR4+ tumor cells were estimated by two board-certified hematopathologists.

### Statistical analysis

A sample size calculation revealed that, to detect a sensitivity difference of 10% (given 14% discordant findings) between [^68^Ga]Pentixafor-PET and [^18^F]FDG-PET with a power of 80% (alpha, 5%; two-sided), and assuming an average of five regions with lymphoma involvement per patient and a dropout rate of 10%, a total number of 96 matched regions with lymphoma involvement, and thus, 22 patients would be needed.

Rates of agreement of [^68^Ga]Pentixafor-PET and [^18^F]FDG-PET with the reference standard, and among the two PET examinations, were calculated *per region*. General Estimation Equations (GEE) that take multiple measurements per patient into account were used for comparison of [^68^Ga]Pentixafor-PET and [^18^F]FDG-PET sensitivities and positive predictive values (PPV). SUVs and TBRs of all lesions were compared between [^68^Ga]Pentixafor-PET and [^18^F]FDG-PET using hierarchic linear models with unstructured covariance matrices in order to take multiple measurements per patient into account. Agreement of [^68^Ga]Pentixafor-PET and [^18^F]FDG-PET in terms of Lugano staging (i.e., on a *per patient* level) was also assessed.

For the spleen and the bone marrow, receiver operating characteristic (ROC) curves were used to determine the optimal SUVs and TBRs cut-off values for the detection of lymphoma involvement, and areas under the curve (AUC) and sensitivities, specificities, PPV, and negative predictive values (NPV) were calculated for the metrics with the highest AUCs. Bland-Altman plots were constructed for comparison of the four quantitative metrics measured on [^68^Ga]Pentixafor-PET and [^18^F]FDG-PET: SUV_max_, SUV_mean,_ TBR_liver,_ and TBR_blood._

Two-tailed Pearson correlation coefficients were used to assess the associations between IHC-based percentages of CXCR4+ cells and [^68^Ga]Pentixafor-PET SUV_max_, SUV_mean,_ TBR_liver,_ and TBR_blood_ that were measured in the same anatomic regions where underlying biopsies or subsequent surgery were performed.

The specified level of significance was *P*<0.05 for all tests. All data analyses were performed with the software package SPSS 24.0 (SPSS Inc., Chicago, IL, USA).

## Results

### Patient characteristics

Twenty-two patients (11 women and 11 men; mean age, 70.0±8.5 years; age range, 52-82 years) were enrolled in our study; one with stage I, three with stage II, three with stage III, and 15 with stage IV disease according to clinical records. Mean white blood count (WBC) was 10.5±11.9 (x10^9^/L), and mean lactate dehydrogenase (LDH) was 232.3±86.0 U/L. Two patients showed MCL with blastoid differentiation. According to the MCL International Prognostic Index (MIPI), there were four low risk, 11 intermediate risk, and seven high-risk patients. [^18^F]FDG-PET/MRI in addition to [^68^Ga]Pentixafor-PET/MRI was performed in 19/22 patients.

Lymphoma involvement according to the reference standard was present in a total of 109 anatomic regions of the 22 patients. Nodal disease was observed in 99/109 involved regions, and extranodal disease in 10/109 regions (excluding the bone marrow and spleen): Waldeyer ring, five; stomach, two; and lungs, soft tissues, and other tissues in one case each. The bone marrow was involved in 11/22 patients: histology was available in 18/22, and in 10/11 patients with bone marrow involvement. In the remaining patient with bone marrow involvement, the diagnosis was based on the presence of increased diffuse [^18^F]FDG uptake (> liver) and diffuse high DWI b800 signal (> muscle) as well as diffuse low T1 signal. The spleen was involved in 9/22 patients; the diagnosis was made based on histology in a single case, based on splenomegaly and increased diffuse [^18^F]FDG uptake (> liver) in six cases, and based on increased (multi)focal [^18^F]FDG uptake with MRI correlate in two patients.

In the 19 patients that underwent both [^68^Ga]Pentixafor-PET/MRI and [^18^F]FDG-PET/MRI, 107 regions (nodal and extranodal, excluding bone marrow and spleen) showed lymphoma involvement according to the reference standard, i.e., 11 regions more than the 96 regions required according to the sample size calculation. In these 19 patients, median blood glucose level was 115 mg/dL (6.4 mmol/L), with a range of 89-142 mg/dL (4.9-7.9 mmol/L).

### [^68^Ga]Pentixafor-PET vs. [^18^F]FDG-PET

[^68^Ga]Pentixafor SUV_max_ and SUV_mean_ were 2.49±0.56 and 1.87±0.55 in the mediastinal blood pool, and 2.68±0.96 and 1.60±0.54 in the liver. [^18^F]FDG-PET SUV_max_ and SUV_mean_ were 2.2±0.61 and 1.70±0.40 in the mediastinal blood pool, and 3.04±0.78 and 2.34±0.83 in the liver.

**Figure 1:**
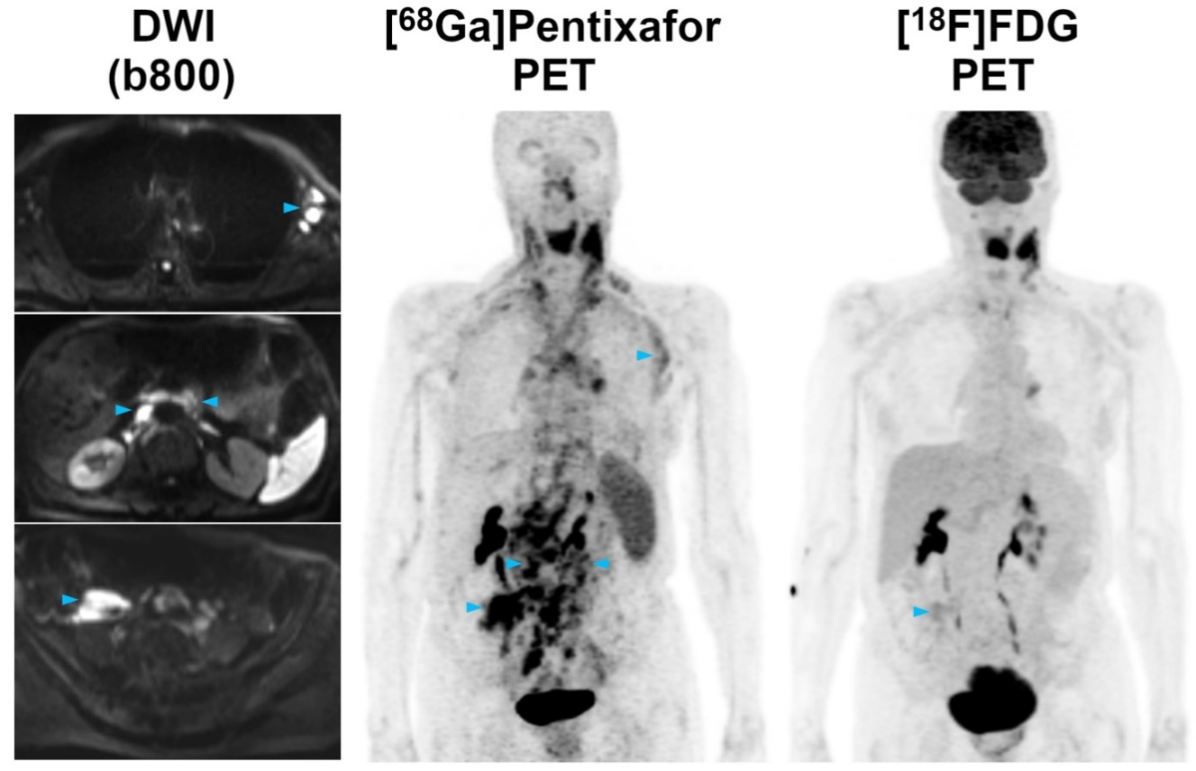
Pre-therapeutic [^68^Ga]Pentixafor-PET/MRI and [^18^F]FDG-PET/MRI of an 82-year-old female MCL patient. [^68^Ga]Pentixafor-PET detects considerably more nodal lymphoma manifestations than [^18^F]FDG-PET, and stronger tracer uptake in several lesions/regions. All lymph nodes with focal increased [^68^Ga]Pentixafor uptake corresponded to clearly enlarged lymph nodes on diffusion-weighted MRI (DWI). Representative examples of such [^68^Ga]Pentixafor-PET-positive lesions with no or low [^18^F]FDG uptake, but obvious lymphadenopathy on DWI, are marked by blue arrowheads (left axially, retroperitoneal/periaortic, and right pelvic regions).

**Figure 2:**
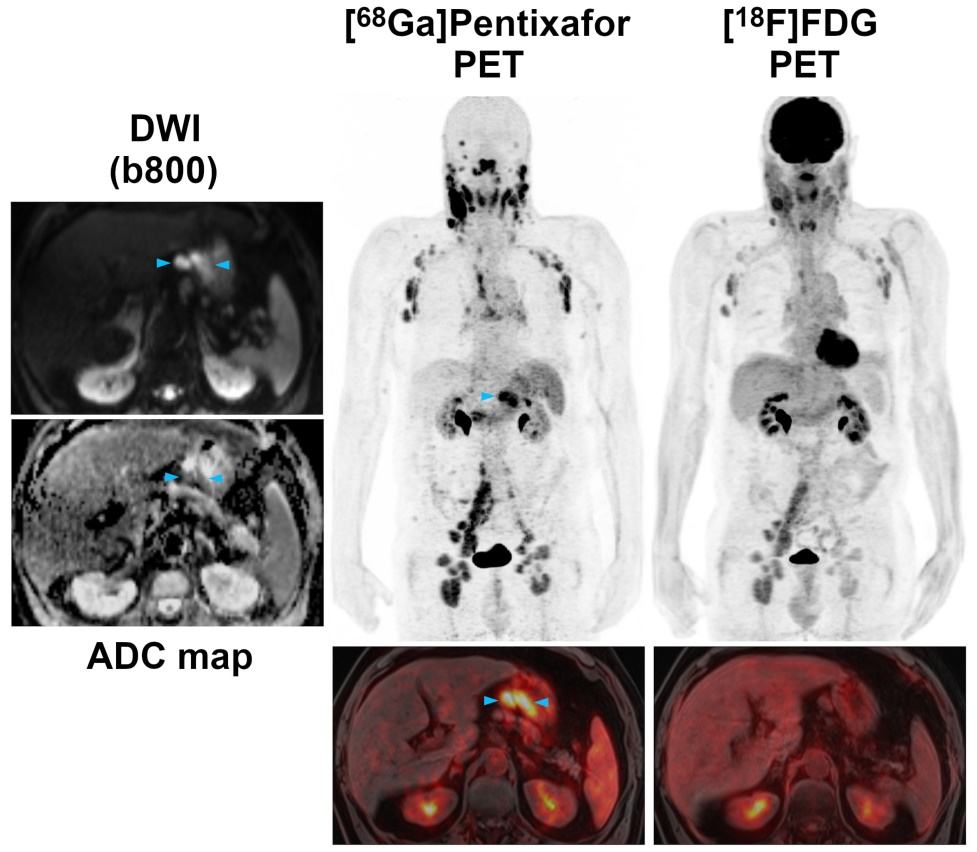
Pre-therapeutic [^68^Ga]Pentixafor-PET/MRI and [^18^F]FDG-PET/MRI of a 61-year-old male MCL patient. While the majority of supra- and infradiaphragmatic nodal lymphoma manifestations show both an increased [^68^Ga]Pentixafor and [^18^F]FDG uptake, the extranodal gastric lymphoma manifestation and an adjacent enlarged lymph node (blue arrowheads) are only positive on [^68^Ga]Pentixafor-PET, but not on [^18^F]FDG-PET. Diffusion-weighted MRI (DWI) confirmed the presence of both lesions, which show a low signal on the apparent diffusion coefficient (ADC) map, reflecting the high cell density typically observed in lymphomas.

In the 19 patients who underwent PET/MRI with both tracers, *per region* agreement between [^68^Ga]Pentixafor-PET (positive in 114 regions) and [^18^F]FDG-PET (positive in 85 regions) was 74.6%, excluding the bone marrow and spleen. Of the 85 [^18^F]FDG-PET positive regions, six (7.1%) showed uptake not higher than the liver background in 4/19 (21.1%) patients. Compared to the reference standard, [^68^Ga]Pentixafor-PET showed a significantly higher sensitivity (100.0%; CI, 100.0-100%) than [^18^F]FDG-PET (75.2%; CI, 66.3-82.3%) (*P*<0.001) (Figures 1 and 2). With seven false-positive regions, PPV was slightly, but not significantly (*P*=0.21), lower for [^68^Ga]Pentixafor-PET (94.0%; CI, 87.9-97.1%) than for [^18^F]FDG-PET (96.5%; CI, 89.6-98.9%), which was false-positive in only two regions.

**Figure 3:**
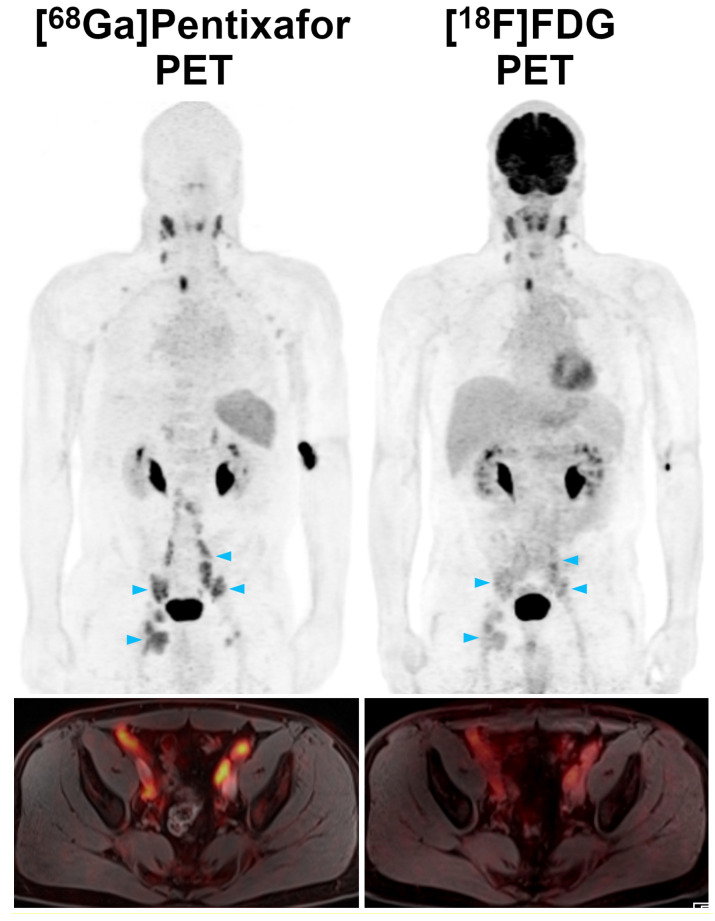
Pre-therapeutic [^68^Ga]Pentixafor-PET/MRI and [^18^F]FDG-PET/MRI of a 52-year-old male MCL patient. Several infradiaphragmatic nodal lymphoma manifestations (blue arrowheads) that show strong [^68^Ga]Pentixafor uptake on PET (top) and fused color-coded PET/MRI (bottom) show only moderate-to-low [^18^F]FDG uptake that does not, or only slightly, exceed the [^18^F]FDG uptake of the liver. Consequently, measured tumor-to-background ratios (TBR_blood_, TBR_liver_) are higher for [^68^Ga]Pentixafor-PET than for [^18^F]FDG-PET.

**Figure 4:**
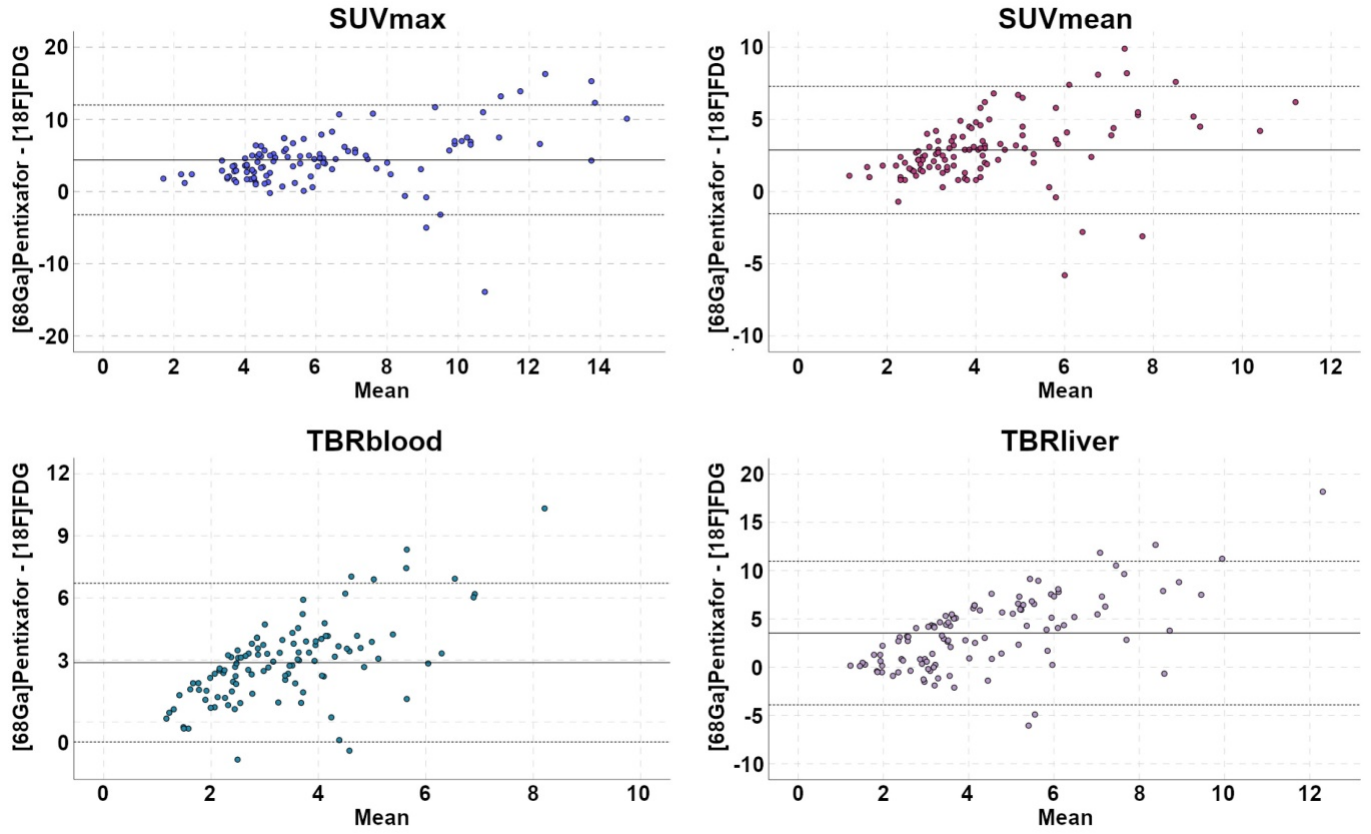
Bland-Altman plots illustrate paired differences between [^68^Ga]Pentixafor-PET and [^18^F]FDG-PET standardized uptake values (SUV) and tumor-to-background ratios (TBR).

SUVs and TBRs were significantly higher on [^68^Ga]Pentixafor-PET than on [^18^F]FDG-PET by a factor of 2-2.5 (*P*<0.001 in all cases) (Table 1, Figure 2). The greatest difference was observed for TBR_blood_, with a mean of 4.9 for [^68^Ga]Pentixafor-PET and 2.0 for [^18^F]FDG-PET (Table 1, Figures 3 and 4).

Staging according to the Lugano classification did not differ between [^68^Ga]Pentixafor-PET and [^18^F]FDG-PET in a single patient. This was due to the fact that in those patients that showed additional, possibly stage-modifying nodal or extranodal involvement on [^68^Ga]Pentixafor-PET that was not seen on [^18^F]FDG-PET (e.g. gastric involvement in Figure 2), bone marrow involvement was detected by biopsy (i.e., a routine test in MCL), and based on it, these patients were already assigned to stage IV.

### Bone marrow and spleen

Mean [^68^Ga]Pentixafor SUV_max_, SUV_mean_, TBR_blood_ and TBR_liver_ of patients with and without MCL involvement of the bone marrow and the spleen are provided in Table 2.

**Figure 5:**
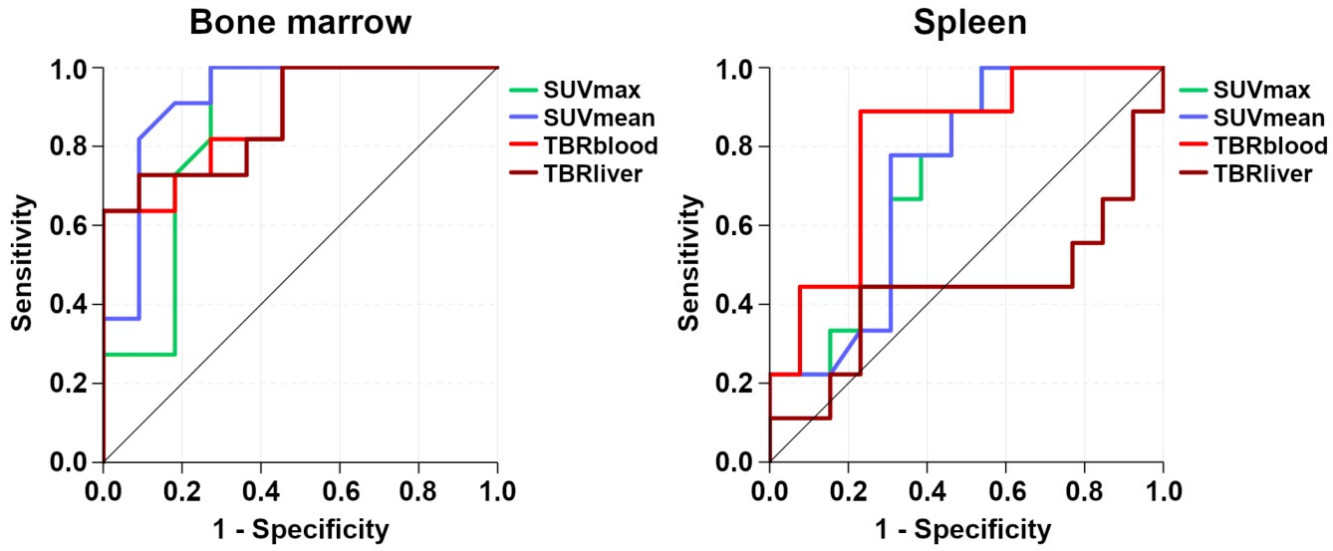
Receiver operating characteristic (ROC) curves that illustrate the respective performances of [^68^Ga]Pentixafor-PET standardized uptake values (SUV) and tumor-to-background ratios (TBR) for detection of bone marrow and splenic involvement.

While all four [^68^Ga]Pentixafor-PET metrics differed significantly between involved and uninvolved bone marrow, SUV_mean_ showed the best performance, with an AUC of 0.92 (Figure 5). Using the calculated optimal SUV_mean_ cut-off value of 2.2, sensitivity was 81.8% (CI, 52.3-94.9%), specificity was 90.9% (CI, 62.3-98.4%), PPV was 90.0% (CI, 59.6-98.2%), and NPV was 83.3% (CI, 55.2-95.3%).

For the spleen, TBR_blood_ was the only [^68^Ga]Pentixafor-PET metric that differed significantly between splenic lymphoma involvement and physiologic uptake, with an AUC of 0.81 (Figure 5). Using the calculated optimal TBR_blood_ cut-off value of 4.0, sensitivity was 88.9% (CI, 56.5-98.0%), specificity was 76.9% (CI, 49.7-91.8%), PPV was 72.7% (CI, 43.4-90.3%), and NPV was 90.9% (CI, 62.3-98.4%).

### IHC and [^68^Ga]Pentixafor metrics

**Figure 6:**
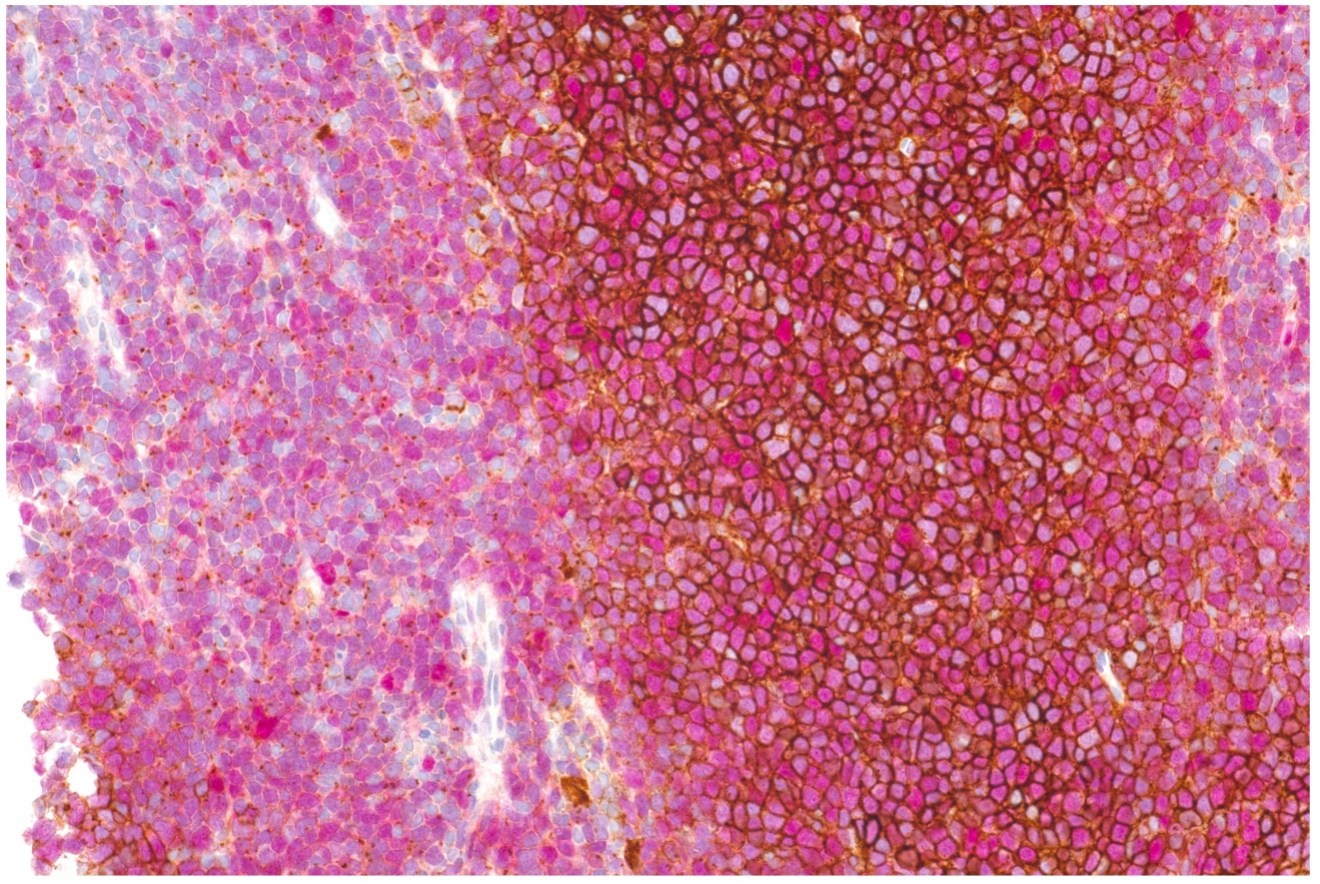
Double staining of CXCR4 (brown) and Cyclin D1 (nuclear, red) showing strong membraneous expression of CXCR4 on MCL cells in the center of neoplastic nodules, whereas in diffuse areas (depicted on the left), mainly dot-like intracellular signals are denoted.

MCL cells from 17/22 patients (total of 24 samples: one sample each in 11 patients; two samples from different anatomic regions in five patients; and three samples from different regions in one patient) were available for analysis (see Figure 6). Twenty samples were Cyclin D1 positive, two partially positive, and two negative. All but two samples were CXCR4 positive, with a mean percentage of CXCR4+ cells of 59.2±31.9% (range, 10-90% in CXCR4+ samples). Pearson correlation coefficients between percentages of CXCR4+ cells and [^68^Ga]Pentixafor-PET metrics were 0.55 for SUV_max_ (*P*=0.006), 0.53 for SUV_mean_ (*P*=0.008), 0.55 for TBR_blood_ (*P*=0.006), and 0.55 for TBR_liver_ (*P*=0.005).

## Discussion

While [^18^F]FDG is the currently recommended radiotracer for PET imaging of MCL 4, it has known shortcomings, in particular that [^18^F]FDG uptake is variable and dependent on tumor grade and aggressiveness 5, and that it cannot reliably capture bone marrow involvement 29-31.

In the present study, we demonstrate that CXCR4 imaging with [^68^Ga]Pentixafor-PET might have potential as an alternative to [^18^F]FDG-PET. Lymphoma detection rates with [^68^Ga]Pentixafor-PET were significantly higher on a *per region* basis than with [^18^F]FDG-PET, with a sensitivity difference of +25%. We did not observe a single anatomic region where lesions were positive on [^18^F]FDG-PET, but negative on [^68^Ga]Pentixafor-PET. While practically all lymphoma manifestations detected by [^68^Ga]Pentixafor-PET, but missed by [^18^F]FDG-PET, were enlarged lymph nodes (Figure 1), gastric involvement was also missed in one patient (Figure 2). A superiority of [^68^Ga]Pentixafor-PET over [^18^F]FDG-PET has previously been reported for a series of 17 patients with Waldenström macroglobulinemia (+65% higher detection rate for [^68^Ga]Pentixafor-PET), and in patients with MALT lymphoma and mycosis fungoides 19-21. Contrary to MCL, [^18^F]FDG-PET is currently not recommended for such indolent lymphomas that show a variable, usually low-level glucose metabolism 4. The latter feature might explain why the superiority of [^68^Ga]Pentixafor-PET over [^18^F]FDG-PET was even greater in Waldenström macroglobulinemia than in MCL 19.

In lymphomas such as MCL, PET is not merely relevant for the assessment of the extent of disease before treatment, but it also provides baseline data for subsequent treatment response assessment. For instance, the Lugano classification criterion for complete remission is a Deauville score of ≤3—i.e., no residual uptake higher than the liver background on post-treatment [^18^F]FDG-PET 4. However, as also shown in Figures 1 and 2, MCL manifestations can show [^18^F]FDG uptake that is not, or only slightly, higher than the liver even before treatment. In such cases, the Lugano response criteria might be difficult to apply. [^68^Ga]Pentixafor uptake, on the other hand, was considerably higher than the blood pool and liver background in all MCL manifestations confirmed by the reference standard (Table 1), and [^68^Ga]Pentixafor TBRs were also markedly higher than for [^18^F]FDG-PET, by a factor of up to 2.5 (for TBR_blood_). Notably, all [^68^Ga]Pentixafor-PET uptake metrics were significantly correlated with CXCR4 expression on MCL cells demonstrated by IHC. This moderate correlation closely resembles previous findings in multiple myeloma xenografts 14.

For the assessment of bone marrow involvement—a criterion for stage IV disease—the Lugano classification currently recommends unilateral iliac crest bone marrow biopsy for staging of all Non-Hodgkin's lymphomas, except diffuse large B-cell lymphoma with PET-positive bone lesions. This is because [^18^F]FDG-PET has limited sensitivity for the detection of especially low-burden infiltration 4, particularly in indolent lymphoma subtypes with a lower [^18^F]FDG uptake. Bone marrow biopsy is associated with considerable pain and anxiety 32, and also carries a small risk of bleeding and infection. Therefore, a non-invasive imaging test to diagnose bone marrow involvement would be desirable. In Waldenström macroglobulinemia and multiple myeloma, the use of [^68^Ga]Pentixafor-PET uptake greater than the liver background as the criterion for bone marrow involvement yielded considerably better detection rates (+35% and +40%, respectively) than [^18^F]FDG-PET 16,19; no comparable visual or quantitative [^68^Ga]Pentixafor-PET criterion is currently established for MCL. Our evaluation shows that several [^68^Ga]Pentixafor-PET metrics have potential in that regard (Table 2). An SUV_mean_ >2.2—a cut-off value that is slightly higher than liver SUV_mean_ values recorded in our study, and that is also in good agreement with previously reported upper limits of physiologic bone marrow [^68^Ga]Pentixafor uptake measurements in pancreatic cancer and MALT lymphoma patients without bone marrow involvement 18—showed a PPV of 90%. Despite these encouraging results, prospective external validation of this cut-off value is clearly needed to determine whether, and down to what percentage of cellular bone marrow infiltration [^68^Ga]Pentixafor-PET could be clinically feasible for the assessment of bone marrow involvement in MCL.

Substantial physiologic tracer uptake in the spleen secondary to accumulation of CXCR4-expressing blood cells is a known feature on [^68^Ga]Pentixafor-PET 18,19,22. However, no cut-off value in terms of [^68^Ga]Pentixafor uptake has, to our knowledge, so far been established in any type of blood cancer to diagnose splenic involvement. For this reason, and because the spleen is rarely biopsied in clinical practice, we used a composite reference standard that included diffuse [^18^F]FDG uptake greater than the liver background 25,26, and splenomegaly with the recommended 13-cm vertical cut-off 4. Because both criteria are, however, not entirely undisputed 25,32, at least two morphological criteria had to be fulfilled to be rated as positive for lymphoma involvement. Contrary to bone marrow, our evaluation of four [^68^Ga]Pentixafor-PET uptake metrics showed that only TBR_blood_ achieved a reasonably good performance for the assessment of lymphoma involvement. With a cut-off value of 4.0, TBR_blood_ showed a high NPV of 91%, indicating low probability of malignancy below that threshold. Notably, in the group without splenic MCL involvement according to the reference standard, splenic [^68^Ga]Pentixafor SUVs were slightly higher than previously reported in small cohorts of patients with different cancers, but without splenic malignancy 18,22. This might possibly have been due to a limited sensitivity of our composite reference standard that did not capture lower level organ infiltration. However, a more in-depth understanding of splenic CXCR4 expression—especially under immunomodulatory therapy with drugs such as ibrutinib—and factors that influence it, is necessary.

Similar to previous observations in MALT lymphoma 20, increased [^68^Ga]Pentixafor uptake in non-enlarged cervical lymph nodes was seen in 6/22 patients of our MCL cohort, leading to a slightly lower PPV compared to [^18^F]FDG-PET. As previously suggested, the most likely explanation for this increased [^68^Ga]Pentixafor uptake is leukocyte activation 20, as CXCR4 mediates B cell homing to secondary lymphatic tissues. It is presently unclear whether this probably physiologic uptake of [^68^Ga]Pentixafor-PET could have actual clinical implications. A simple correlation with the morphological imaging test (computed tomography or MRI) that is part of PET/CT or PET/MRI would aid in distinguishing between reactive/inflammatory lymph nodes and lymphomatous lymph nodes. However, targeted biopsies would nevertheless be necessary to confirm that such small [^68^Ga]Pentixafor-positive lymph nodes are indeed false-positive in all cases (as assumed in the present study), and do not represent “microinfiltration” by MCL.

In addition to the above described, established systemic treatments as well as novel investigational drugs that directly or indirectly target the CXCR4/CXCL12 axis 10-12,34, a theranostic approach using [^177^Lu]Pentixather has shown great promise in multiple myeloma and acute leukemia 35-37. A recent pre-clinical study suggested that a second-generation therapeutic CXCR4 ligand, [^177^Lu]DOTA-r-a-ABA-CPCR4, may provide even better targeting efficiency and tumor retention than [^177^Lu]Pentixather 38. Such a theranostic treatment strategy could possibly also be used in MCL, for instance in patients with no or inadequate response to first line or even second line systemic treatment, or, similar to classic radiotherapy, as an addition to current immuno-chemotherapy regimens. Clinical trials to investigate these options are currently in the planning phase at our institutions.

Our study has several limitations, the most obvious one being the small sample size. Furthermore, a clear limitation is that, since purely study-related biopsies for confirmation of PET-positive lesions would neither be clinically feasible nor ethically justifiable, the established Lugano classification size criteria were applied to MRI to confirm focally increased uptake on PET, due to a lack of other options. While this strategy was necessary for our sample size calculation, and to calculate sensitivity and PPV, morphological criteria are clearly suboptimal for testing the performance of molecular imaging tests such as PET, which are designed to be more sensitive as well as more specific than morphologic imaging tests, detecting the presence of disease ahead of structural changes. For this reason, we refrained from using morphologic criteria to also assess specificity and NPV, because reactive/inflammatory lymph nodes, for instance, in the axilla or groin, frequently exceed 1.5 cm in the long-axis diameter.

## Conclusions

Our findings in a small cohort of MCL patients suggest that [^68^Ga]Pentixafor-PET may become an alternative to [^18^F]FDG-PET in this lymphoma subtype, showing clearly higher detection rates and better tumor-to-background contrast. [^68^Ga]Pentixafor-PET metrics also appear to have potential for the non-invasive assessment of bone marrow involvement, and possibly also involvement of the spleen. However, validation of cut-off values in external cohorts is required before definitive conclusions about the clinical applicability of this imaging test can be drawn.

## Figures and Tables

**Table 1:** Comparison of [^68^Ga]Pentixafor-PET and [^18^F]FDG-PET standardized uptake values (SUV) and tumor-to-background ratios (TBR) in PET-positive lymphoma manifestations (excluding bone marrow and spleen)

	Mean	Standard error	95% Confidence interval	*P* value
**SUV_max_:**				
[^18^F]FDG	4.08	0.26	3.57 - 4.59	<0.001
[^68^Ga]Pentixafor	8.44	0.38	7.68 - 9.19	
**SUV_mean_:**				
[^18^F]FDG	2.80	0.17	2.47 - 3.14	<0.001
[^68^Ga]Pentixafor	5.65	0.24	5.18 - 6.12	
**TBR_blood_:**				
[^18^F]FDG	1.95	0.10	1.75 - 2.15	<0.001
[^68^Ga]Pentixafor	4.85	0.21	4.43 - 5.28	
**TBR_liver_:**				
[^18^F]FDG	2.65	0.16	2.33 - 2.96	<0.001
[^68^Ga]Pentixafor	6.21	0.35	5.52 - 6.90	

**Table 2:** Performance of [^68^Ga]Pentixafor-PET uptake metrics for assessment of bone marrow and spleen lymphoma involvement

	Mean	AUC	Standard error	95% Confidence interval	*P* value
**Bone marrow:**					
SUV_max_	(-): 2.68(+): 3.92	0.85	0.091	0.67 - 1.0	0.006
SUV_mean_	(-): 1.68(+): 2.74	0.92	0.062	0.80 - 1.0	0.001
TBR_blood_	(-): 1.42(+): 2.29	0.88	0.073	0.73 - 1.0	0.003
TBR_liver_	(-): 1.75(+): 2.51	0.88	0.074	0.73 - 1.0	0.003
**Spleen:**					
SUV_max_	(-): 6.89(+): 9.16	0.74	0.11	0.53 - 0.94	0.07
SUV_mean_	(-): 5.10(+): 6.73	0.74	0.11	0.52 - 0.94	0.07
TBR_blood_	(-): 3.72(+): 4.90	0.81	0.09	0.63 - 1.0	0.02
TBR_liver_	(-): 4.98(+): 5.20	0.44	0.14	0.16 - 0.71	0.62

(-) and (+), negative and positive for lymphoma according to reference standard

## Abbreviations

MCL: mantle cell lymphoma
[^18^F]FDG: 2-deoxy-2-[^18^F]fluoro-D-glucose
ADC: apparent diffusion coefficient
AUC: area under the curve
DWI: diffusion-weighted imaging
EPI: echo-planar imaging
FOV: field of view
GEE: general estimation equations
HASTE: half-Fourier acquisition single-shot turbo spin-echo
IHC: immunohistochemistry
MRI: magnetic resonance imaging
NPV: negative predictive value
PET: positron emission tomography
PPV: positive predictive value
ROC: receiver operating characteristics
SUV: standardized uptake value
TBR: tumor-to-background ratio
TE: echo time
TR: repetition time
VIBE: volume-interpolated breath-hold
